# Secondary messenger signalling influences *Pseudomonas aeruginosa* adaptation to sinus and lung environments

**DOI:** 10.1093/ismejo/wrae065

**Published:** 2024-04-22

**Authors:** Dilem Ruhluel, Lewis Fisher, Thomas E Barton, Hollie Leighton, Sumit Kumar, Paula Amores Morillo, Siobhan O’Brien, Joanne L Fothergill, Daniel R Neill

**Affiliations:** Department of Clinical Infection, Microbiology and Immunology, University of Liverpool, Ronald Ross Building, 8 West Derby Street, Liverpool, United Kingdom; Wellcome Sanger Institute, Wellcome Genome Campus, Cambridge, United Kingdom; Division of Molecular Microbiology, University of Dundee, Dow Street, Dundee, United Kingdom; Department of Clinical Infection, Microbiology and Immunology, University of Liverpool, Ronald Ross Building, 8 West Derby Street, Liverpool, United Kingdom; Division of Molecular Microbiology, University of Dundee, Dow Street, Dundee, United Kingdom; Department of Clinical Infection, Microbiology and Immunology, University of Liverpool, Ronald Ross Building, 8 West Derby Street, Liverpool, United Kingdom; Department of Microbiology, Moyne Institute of Preventive Medicine, Trinity College, Dublin, 2, Ireland; Department of Clinical Infection, Microbiology and Immunology, University of Liverpool, Ronald Ross Building, 8 West Derby Street, Liverpool, United Kingdom; Division of Molecular Microbiology, University of Dundee, Dow Street, Dundee, United Kingdom

**Keywords:** Pseudomonas aeruginosa, respiratory tract infection, within-host evolution, cyclic-di-GMP, cystic fibrosis

## Abstract

*Pseudomonas aeruginosa* is a cause of chronic respiratory tract infections in people with cystic fibrosis (CF), non-CF bronchiectasis, and chronic obstructive pulmonary disease. Prolonged infection allows the accumulation of mutations and horizontal gene transfer, increasing the likelihood of adaptive phenotypic traits. Adaptation is proposed to arise first in bacterial populations colonizing upper airway environments. Here, we model this process using an experimental evolution approach. *Pseudomonas aeruginosa* PAO1, which is not airway adapted, was serially passaged, separately, in media chemically reflective of upper or lower airway environments. To explore whether the CF environment selects for unique traits, we separately passaged PAO1 in airway-mimicking media with or without CF-specific factors. Our findings demonstrated that all airway environments—sinus and lungs, under CF and non-CF conditions—selected for loss of twitching motility, increased resistance to multiple antibiotic classes, and a hyper-biofilm phenotype. These traits conferred increased airway colonization potential in an *in vivo* model. CF-like conditions exerted stronger selective pressures, leading to emergence of more pronounced phenotypes. Loss of twitching was associated with mutations in type IV pili genes*.* Type IV pili mediate surface attachment, twitching, and induction of cAMP signalling. We additionally identified multiple evolutionary routes to increased biofilm formation involving regulation of cyclic-di-GMP signalling. These included the loss of function mutations in *bifA* and *dipA* phosphodiesterase genes and activating mutations in the *siaA* phosphatase. These data highlight that airway environments select for traits associated with sessile lifestyles and suggest upper airway niches support emergence of phenotypes that promote establishment of lung infection.

## Graphical Abstract

**Graphical Abstract:**
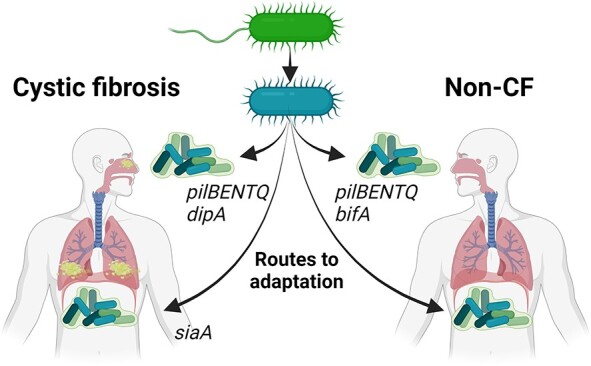

## Introduction

Chronic respiratory tract infection with *Pseudomonas aeruginosa* is associated with a process of within-host adaptation that leads to the emergence of one or more of a characteristic set of bacterial phenotypes, including slow growth, increased biofilm formation, and reduced motility [1, 2]. These traits contribute to the enhanced antimicrobial resistance that is a feature of chronic *P. aeruginosa* infection [3]. Our understanding of *P. aeruginosa* adaptation and evolution within the airways comes from longitudinal sampling of sputum in chronically infected individuals, especially those with cystic fibrosis (CF) [2, 4, 5]. Less is known about adaptation in the context of other respiratory conditions, such as chronic obstructive pulmonary disease (COPD) or non-CF bronchiectasis (NCFB), despite the prevalence of *P. aeruginosa* infections in these patient groups [6, 7]. Biofilm-promoting mutations have been identified in isolates from non-CF bronchiectasis [1, 8] and pathoadaptive mutations commonly associated with CF isolates have also been described in those from COPD [9].

Transmissible lineages of host-adapted *P. aeruginosa* circulate amongst those with impaired airway defences, but environmental isolates are also capable of establishing respiratory tract infection and the phylogeny of *P. aeruginosa* causing infection in people with CF largely overlays that of the species more broadly [10]. Data from both clinical and experimental studies suggest that, following initial colonization of the respiratory tract by an environmental *P. aeruginosa* isolate, a period of rapid adaptation to host conditions takes place within upper airway niches, principally the paranasal sinuses, before the onset of chronic lung infection [11–14]. Upper respiratory tract environments act as a protective niche, with little immune surveillance and a nutrient landscape supporting a quiescent lifestyle [14]. Paired upper and lower airway isolates from individual patients are often genetically indistinguishable [15, 16], but little is known about the drivers of the adaptive evolutionary processes taking place in the sinuses, prior to establishment of lung infection.

Here, we used a suite of chemically defined media, designed to be reflective of airway conditions [17], to investigate processes by which *P. aeruginosa* adapts to respiratory environments. We separately examined upper and lower airway conditions, with or without the addition of CF-specific factors, to enable us to explore the relative contribution of niche and of disease condition to adaptive evolutionary processes. We demonstrate that airway environments select for a common set of phenotypes, with the same traits emerging under both upper and lower airway conditions. CF-like conditions exert stronger selective pressures than those associated with other respiratory syndromes. We describe multiple evolutionary routes to enhanced biofilm formation, through modulation and decoupling of cAMP and c-di-GMP signalling.

## Materials and Methods

### Ethics statement

Animal work was conducted at the University of Liverpool, under UK Home Office project licence PP2072053 and with prior approval from the local Animal Welfare Ethical Review Board. The principles of the Declaration of Helsinki were observed throughout. Mice were housed in individually ventilated cages, with access to food and water *ad libitum*. Environmental enrichment was provided, and mice were acclimatized to the animal unit for 7 days before use. Mice were randomly allocated to cages by staff with no role in study design. Individual mice were considered as the experimental unit. Sample sizes, controls, and statistical analyses are detailed in figure legends. No samples were excluded from analyses.

### Bacteria

All experimentally evolved populations were derived from a PAO1 isolate from the *P. aeruginosa* international reference panel [18]. PAO1 transposon library was obtained from the Manoil Lab (University of Washington, USA) [19]. Bacteria were grown on Tryptic Soy agar (TSA) plates inoculated from frozen stocks and incubated for 18 h at 37°C. For liquid culture, a sweep of colonies was resuspended in 5–10 ml Luria-Bertani (Miller) (LB) broth before incubation at 37°C, shaking at 180 rpm in a Stuart SI500 (Stuart Equipment, USA) incubator.

### Airway-mimicking media

Sinus media (SM), lung media (LM), CF sinus media (CFSM), and CF lung media (CFLM) were prepared as previously described [17]. CF media differ from SM and LM due to higher concentrations of mucin, DNA, free amino acids and host-derived antimicrobials. Glucose is added to reflect the effects of CF-associated diabetes and bile salts are introduced to capture effects of gastro-oesophageal reflux disease, a common CF co-morbidity. SM has lower protein and polyamine concentrations than LM. Non-CF airway media (SM, LM) are Newtonian fluids, while CF media (CFSM, CFLM) are more viscous [17]. All media support the growth of planktonic bacteria as well as suspended aggregates and biofilms attached to the vessel wall. SM are cultured at 34°C, 0% CO_2_, LM cultures were incubated at 37°C, 5% CO_2_. Gentle shaking (150 rpm) was employed during culture. Freshly prepared media were divided into 50 ml single use aliquots and stored at −80°C until use.

### Experimental evolution of PAO1 in airway-mimicking media

PAO1 was streaked onto TSA and incubated at 37°C for 18 h. To obtain five independent founder populations for experimental evolution, five individual colonies were selected, and each inoculated in 10-ml LB and cultured for 18 h at 37°C. Cultures were adjusted to OD_600_ 0.05 ± 0.01, and 200 μl of each of the five independent cultures was added to four universal glass tubes containing 10 ml of SM, LM, CFSM, and CFLM, respectively. This resulted in 20 independently evolving populations (5 per media). After 48 h under niche-appropriate conditions, cultures were disrupted by addition of 10-ml Sputasol (Thermo Fisher) and thoroughly homogenized to ensure the mixing of planktonic populations and those in pellicle biofilms or attached to the wall of culture vessels. Subculture into fresh media was then performed by transferring 100 μl (1%) into 10-ml airway-mimicking media. Each population was passaged 20 times, giving a total evolution time of 40 days. Every fifth passage, cultures were plated on Tryptone Congo red/Coomassie blue Agar (TCCA), prepared by mixing 20-mg/l Coomassie blue (Sigma-Aldrich), 40-mg/l Congo red (Sigma-Aldrich), 10-g/l Tryptone (Sigma-Aldrich), and 12-g/l Bacto agar (Fisher-Scientific) in distilled water and autoclaving at 121°C for 15 min. Plates were incubated at 37°C for 24 h and for a further 48 h at room temperature to allow the colonies to uptake the dyes. Bacterial cultures from every fifth passage were stored on cryovial beads (Pro-Lab) for further use. Cultures were confirmed free of contamination at each transfer by plating onto agar.

### Growth curves

Evolved populations (passage 20) and ancestors were cultured overnight in 5-ml LB and adjusted to OD600 0.05 ± 0.01 in LB, and 200-μl/well cultures were incubated in U-bottomed 96-well plates (Greiner) for 24 h. OD600 was measured at 10-min intervals using a Fluostar Omega microplate reader (BMG Labtech), with 15 s shaking prior to each measurement. LB-only controls were included in all assay runs, to confirm sterility of culture media. Growth curves were analyzed using the GrowthcurveR package in R studio [20]. AUC_l, generation time, and carrying capacity were calculated using 24-h growth curve data.

### DNA sequencing and variant calling

Populations of the five PAO1 ancestor colonies and passage 1, 5, 10, 15, and 20 populations from each condition were grown overnight in 5-ml LB and DNA extracted using the Wizard Genomic DNA Purification Kit (Promega), according to manufacturer’s instructions. DNA sample concentration and purity were determined by Nanodrop and Qubit (Life Technologies). Samples at 30 ng/μl in nuclease-free water were submitted to MicrobesNG (Birmingham, UK) for library preparation and short-read sequencing with 30× coverage, using the NovaSeq 6000 platform (Illumina) with 2 × 250 base pair kits. Reads were mapped against a PAO1 reference genome (GCF_000006765.1) and variants were called using Breseq [21], in population mode, using default parameters. Variants identified in the five ancestor PAO1 populations were excluded from subsequent analysis.

### Competition assays

Overnight cultures of a gentamicin-resistant PAO1, labelled using a mini-Tn*7* transposon [22], the ancestor PAO1 populations, and endpoint (passage 20) experimentally evolved populations were diluted to OD600 of 0.05 ± 0.01. The five independent populations from each condition were pooled together (e.g. Ancestor populations 1–5 were pooled, SM populations 1–5 were pooled), yielding 5 pooled cultures (ancestor PAO1, SM-evolved, LM-evolved, CFSM-evolved, CFLM-evolved), and 50 μl of each pooled culture and 50 μl of the gentamicin-resistant PAO1 culture were then mixed and diluted 1:100 in the appropriate media. Competitions were gentamicin-resistant PAO1 vs PAO1 ancestors in LB, gentamicin-resistant PAO1 vs SM-evolved populations in SM, gentamicin-resistant PAO1 vs LM-evolved populations in LM, gentamicin-resistant PAO1 vs CFSM-evolved populations in CFSM, and gentamicin-resistant PAO1 vs CFLM-evolved populations in CFLM. Input populations (time zero) and 24-h cultures under niche-specific conditions were serially diluted onto non-selective (LA) and selective (LA + 10-μg/ml gentamicin) agar. Individual (non-competing) cultures of each strain or population were included in each experiment to confirm appropriate fitness of starting cultures. Plates were incubated at 37°C overnight and colony forming units determined. The number of colonies on the gentamicin-containing plates were subtracted from the colony count from the no antibiotic plates to estimate the colony number of each strain. Total population density change per population per media was estimated as the Malthusian growth parameter (*m*): ln (final density/start density). The result of the gentamicin-resistant PAO1 vs PAO1 ancestor competition was used to quantify the fitness defect associated with carrying the gentamicin resistance cassette. This was measured as a fitness coefficient (*w*) for the gentamicin-resistant PAO1 of 0.61. The results of competition assays between evolved populations and the gentamicin-resistant PAO1 were therefore adjusted by a factor of 0.61. Fitness coefficients (*w*) were determined for pooled populations from each of the four media vs the ancestor in that same media. Individual competitions vs gentamicin-resistant PAO1 were additionally undertaken for each independently evolved population, both in the media within which they were evolved and in nutrient broth (LB). Data were processed as described for the pooled population competitions.

### Mouse inhalation infection model

Female BALB/c mice (7–8 weeks old) were purchased from Charles River UK. Animals were anaesthetized with O_2_/isoflurane and infected intranasally with a fresh, mid-log phase dose of 2 × 10^6^ colony forming units in 50-μl PBS of PAO1 ancestor population 4 or its endpoint populations evolved under each of the four media conditions. Mice did not develop visible disease signs and were culled at predetermined times post infection (Days 1, 3, and 5), by cervical dislocation. Lungs and upper respiratory tract tissue (nasopharynx and sinuses) were removed, processed with a hand-held tissue homogenizer, and serially diluted onto Pseudomonas selective agar for determination of infection burden.

### Twitching motility assay

Colonies were stabbed to the bottom of an LB agar plate, using a pipette tip, and incubated for 24 h. A sterile pipette tip was stabbed to the bottom of a separate plate, as a negative control. Agar was removed with forceps and 10-ml 0.25% (w/v) crystal violet (CV) (Sigma-Aldrich) added to plates for 30 min, staining the area of bacterial growth. CV was removed, and plates rinsed with water. The diameter of bacterial growth was measured at the widest point. Diameters <5 mm were considered twitching impaired.

### Surface-attached biofilm assay

Overnight liquid cultures were diluted 1:100 in airway-mimicking media and 180 μl was added to U-bottomed polystyrene 96-well microtiter plate (Greiner). To minimize edge-effects, perimeter wells were filled with sterile PBS. Airway-mimicking media alone was used as a negative control. Following 3 days static growth under niche-specific conditions, supernatant (containing non-adhered cells) was removed and plates were rinsed with PBS, and 200 μl of CV (0.5%) was added to each well and incubated for 20 mins before washing under running water. CV was solubilized in 200 μl 100% ethanol (Sigma-Aldrich) and incubated for 30 min. Absorbance was measured at OD_600_ using a BMG plate reader. Comparable biofilm phenotypes for our in-house PAO1 and the PAO1 from the transposon library were confirmed, prior to the use of transposon mutants (Supplementary Fig. 1, see online supplementary material for a colour version of this figure).

### Pellicle biofilm assay

Overnight LB liquid cultures at OD_600_ 0.05 ± 0.01 were diluted in airway-mimicking media (1:100) to a volume of 10 ml in glass universal tubes. Airway-mimicking media alone was used as a negative control. Cultures were incubated under niche-specific conditions for 3 days, shaking at 75 rpm, after which biofilms were disrupted using 250 μl of 100 mg/ml cellulase (diluted in 0.05 M citrate buffer [9.6 g/l Citrate.H_2_0 (VWR)] in water, pH adjusted to 4.6 with NaOH) and incubated under oxic conditions, 37°C, shaking at 150 rpm, for 1 h. Manual pipetting ensured complete disruption of biofilms before transfer to 96-well plates. Metabolic activity was measured by addition of 10 μl of 0.02% (v/v) resazurin (Sigma-Aldrich) in distilled water and incubation for 2 h at niche-specific temperatures, shaking at 150 rpm. Fluorescence was measured at excitation wavelength 540 nm and emission wavelength 590 nm in a Fluostar Omega microplate reader. Comparable biofilm phenotypes for our in-house PAO1 and the PAO1 from the transposon library were confirmed, prior to the use of transposon mutants (Supplementary Fig. 1, see online supplementary material for a colour version of this figure).

### Gene expression analysis

Bacteria were grown until early stationary phase in LB (12 h) or CFLM (18 h). TRI reagent (ZYMO Research) was added and incubated for 5–10 min at room temperature. Bacteria were pelleted by centrifugation and RNA isolated using the Direct Zol RNA Microprep kit (ZYMO Research), according to the manufacturer’s instructions with DNase1 digestion. RNA was quantified at OD_260_ using the NanoDrop8000 UV–vis Spectrophotometer (Thermo Scientific). Purity was determined by 260/280 nm ratio (target 1.8–2.0). First-strand cDNA synthesis was performed using iScript cDNA synthesis kit (BIO-RAD: 1708891), and 2.5-ng RNA was incubated in a thermocycler (Applied Biosystem) for 5 min at 25°C, 30 min at 42°C, and then 5 min at 85°C. A no reverse transcriptase control was included for assessment of DNA contamination. cDNA was stored at −20°C until further use.

qRT-PCR was performed in duplicate using the GoTaq® qPCR Master Mix (Promega), as per manufacturer’s instructions. Reactions contained 2-μl cDNA and 0.2-μM forward and reverse primers (Eurofins). Primer sequences: *siaA_F*_CTCCCACCACTACTACTTCAAC, *siaA_R*_TGTTGCGCAGGGTATTGA, *rpoD_F*:GGGCGAAGAAAGGAAATGGT, and *rpoD_R*_CAGGTGGCGTAGGTGCAGA. Template-free and DNA polymerase-free controls were included in each assay run. PCRs were performed on the BioRad CFX Connect Real Time PCR System (BIORAD) using MicroAmp™ Optical 96-Well Reaction Plates (Applied Biosystems) under the following conditions; 2 min at 95°C followed by 40 cycles of 15 s at 95°C and 1 min at 60°C. Analysis of relative gene expression of evolved populations vs ancestor in airway-mimicking media or LB was performed using the 2^−ΔΔCt^ relative quantification method.

### Antibiotic disc diffusion assay

Eighteen hours bacterial cultures in LB were adjusted to OD600 0.5. Muller Hinton agar (Sigma-Aldrich) plates were inoculated with by swabbing in three directions with a cotton swab. Antibiotic discs were applied within 15 min of inoculation and incubated at 37°C for 18 h. Inhibition zone was determined by measuring the halo diameter around the disc.

### Statistics

Unless otherwise stated, data were analyzed by ANOVA with post-hoc analysis and correction for multiple comparison testing. Ancestor populations were included in analysis and used as the comparator group for post-hoc testing.

## Results

### Experimental evolution of *P. aeruginosa* in airway-mimicking media

We developed bacterial growth media reflective of upper airway conditions (SM) and those of the lung (LM) [17]. Both media can be modified by addition of CF-specific factors, including bile salts and elevated concentrations of sugars and host-derived antimicrobials, to give CFSM and CFLM. To explore the process of adaptation to airway environments, we serially passaged a non-CF, non-airway *P. aeruginosa* isolate (PAO1) in these four different media conditions. This was performed in parallel, with five cultures prepared from five individual colonies, yielding five populations each of SM-, LM-, CFSM-, and CFLM-passaged PAO1. Each population was cultured for a total of 40 days in airway-mimicking media, with transfer of 1% of the population into fresh media, every 48 h.

**Figure 1:**
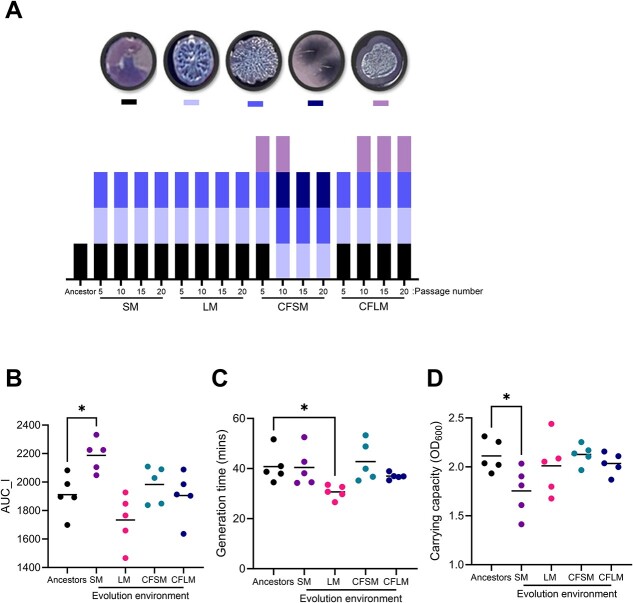
Experimental evolution of *P. aeruginosa* PAO1 in airway-mimicking media; (A) colony morphologies of PAO1 on TCCA agar. Five distinct colony morphologies were observed during experimental evolution in airway-mimicking media, and these are represented as coloured blocks, with the ancestral PAO1 morphotype in black. Morphotypes were assessed at passages 5, 10, 15, and 20, corresponding to Days 10, 20, 30, and 40 of the experiment, and the growth dynamics of end-point (passage 20) populations, evolved under each condition, were determined in LB and area under the logistic curve (B), generation time (C), and carrying capacity (D) quantified using the GrowthCurveR package in R; each data point represents an individually evolved population, with the five ancestral PAO1 colonies shown in black; statistical analysis was by one-way ANOVA with Dunnett’s multiple comparisons test; ^*^ = *P* < .05; data in (B–D) are the per-population average of three independent experiments.

Samples were taken at 10-day intervals and plated onto taurocholate cycloserine cefoxitin agar. The emergence of novel colony morphologies was apparent from Day 10 onward (Fig. 1A). Two wrinkly colony morphotypes were recovered from all four media conditions. One colony type was found only in CF-like conditions (CFSM and CFLM) and one was unique to conditions mimicking the CF sinus (CFSM).

**Figure 2:**
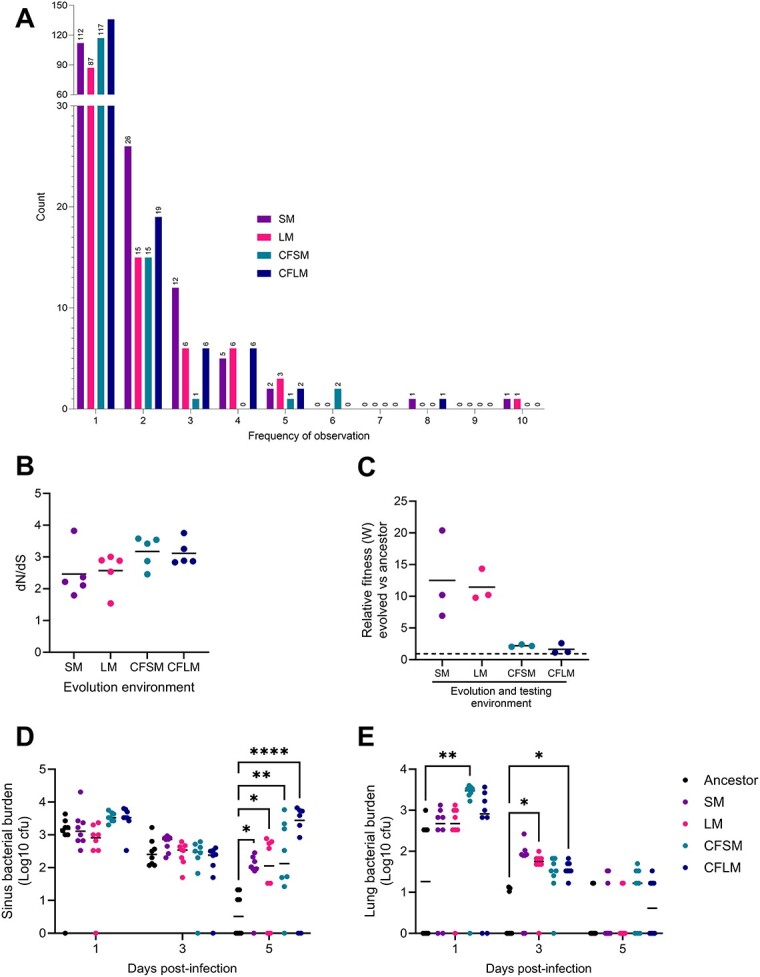
Evidence of niche-adaptation in experimentally evolved PAO1; short-read illumina sequencing of experimentally evolved populations was undertaken on populations at passages 1, 5, 10, 15, and 20 (5 populations each, under 4 conditions, at 5 time points, plus 5 ancestor PAO1 colonies, giving 105 samples), and reads were mapped to an annotated PAO1 genome (GCF_000006765.1) and variants identified using Breseq; variants present in the ancestor colonies were excluded from subsequent analysis; (A) the number of variants present at a frequency of >10% in individual populations and the number of times each unique variant was observed; (B) population- and environment-specific ratios of non-synonymous to synonymous mutations (dN/dS) as an indicator of selection; each data point is an individual population, and (C) relative fitness of passage 20 populations vs PAO1 tagged with a gentamicin resistance cassette, determined by a 24-h competition assay using the media in which each set of populations had been evolved; for each environmental condition, the five separately evolved populations were pooled, and relative fitness calculations were performed by calculating the Malthusian parameter (growth rate; *m*) for each competitor as ln(final density/starting density) and by taking the ratio between PAO1 and evolved populations (m_PAO1/m_population) to get a fitness coefficient (*W*). *W* > 1 (above dashed line) represents enhanced fitness relative to the ancestor, under the conditions tested, and calculations were adjusted to account for the fitness disparity between PAO1 and gentamicin-resistant PAO1. Data are pooled from three independent experiments. (D, E) colony forming units recovered from (D) upper respiratory tract tissue (nasopharynx and paranasal sinuses) and (E) lungs of BALB/c mice infected with PAO1 ancestor 4 or derived populations evolved in each of the four airway-mimicking media; data are from a single experiment and each data point represents an individual mouse; statistical analysis are by two-way ANOVA with Dunnett’s multiple comparison test, with the ancestor set as the comparator group for each experimentally evolved population; ^*^ = *P* < .05, ^*^^*^ = *P* < .01, ^*^^*^^*^^*^ = *P* < .0001.

Growth profiling of the 40 day evolved populations was undertaken in standard laboratory media (LB) (Fig. 1B–DSupplementary Fig. 2, see online supplementary material for a colour version of this figure). Populations displayed altered growth characteristics, relative to the ancestor PAO1 (one-way ANOVA, *P* = .0022, *F* = 6.1, DF = 4, 20). Populations evolved under non-CF sinus conditions (SM) demonstrated moderately increased total productivity, as determined by area under the logistic curve analysis (*P* = .0269 vs ancestor) (Fig. 1B), whilst those evolved under non-CF lung conditions (LM) had a shortened doubling time (*P* = .0417) (Fig. 1C). SM-evolved populations had reduced maximum culture density (carrying capacity) in LB (*P* = .0329) (Fig. 1D).

### Increased environmental fitness of airway-adapted PAO1

We undertook whole genome population sequencing analysis of airway-adapted PAO1 and the ancestor isolates from which they were derived. Short-read sequences of the five ancestral PAO1 colonies and the passage 1, 5, 10, 15, and 20 populations were mapped against a PAO1 genome assembly (GCF_000006765.1) [23]. Single nucleotide polymorphisms, insertions, and deletions were identified using Breseq [21]. After elimination of variants present in the ancestor populations, we identified 483 unique mutations, present at a >10% frequency in individual populations, across the 20 experimentally evolved populations and the 5 time points (Fig. 2A). Most mutations were observed only once—that is, in one population at one passage—but 62 were observed three times or more, with 21 becoming fixed in one or more populations (Supplementary Dataset 1). The mean ratio of non-synonymous to synonymous mutations (dN/dS) was above 2 for all conditions, indicating selection, with the highest ratios observed under CF-like conditions (Fig. 2B).

To determine whether observed changes altered environment-specific fitness, pools of the five independent populations from each condition were competed against the ancestor PAO1 in the media within which they had been evolved. All population pools showed increased fitness (*W* > 1) within their respective media, with populations evolved in the two non-CF environments showing the most pronounced fitness changes (Fig. 2C) (SM *P* = .0235, LM *P* = .0010, CFSM *P* = .0002, CFLM *P* = .1275 vs *W* = 1 in one-tailed *t*-test). In competition experiments performed with each independent population, 13/20 showed significantly enhanced fitness relative to the ancestor PAO1 in airway-mimicking media and 16/20 had a mean relative fitness greater than 1 (Supplementary Fig. 3A, see online supplementary material for a colour version of this figure). This fitness advantage was either lost or severely diminished when competitions were performed in nutrient broth (Supplementary Fig. 3B, see online supplementary material for a colour version of this figure).

We assessed the potential of evolved populations to colonize sinus and lung, in a mouse model of *P. aeruginosa* infection that does not induce acute systemic disease [13]. As ethical considerations precluded testing all 20 populations, we assessed only those derived from one of the original five PAO1 colonies. Endpoint (passage 20) populations evolved in each of the four environments showed an enhanced ability to colonize both sinuses and lung (Fig. 2D and E). In sinuses, all populations demonstrated enhanced colonization density at Day 5 postinfection (Fig. 2D) (two-way ANOVA, *P* = .0190, *F* = 3.1, DF = 4, 105), while in lungs, the rate of bacterial clearance was more rapid for the ancestor as compared with the evolved populations (Fig. 2E) (two-way ANOVA, *P* = .0016, *F* = 4.7, DF = 4, 105). In line with previous observations using airway-adapted *P. aeruginosa* in this murine infection model, infection was largely cleared from the lungs by Day 5 postinfection but persisted in nasopharynx [13].

### Routes to adaptation in airway-adapted PAO1

**Figure 3:**
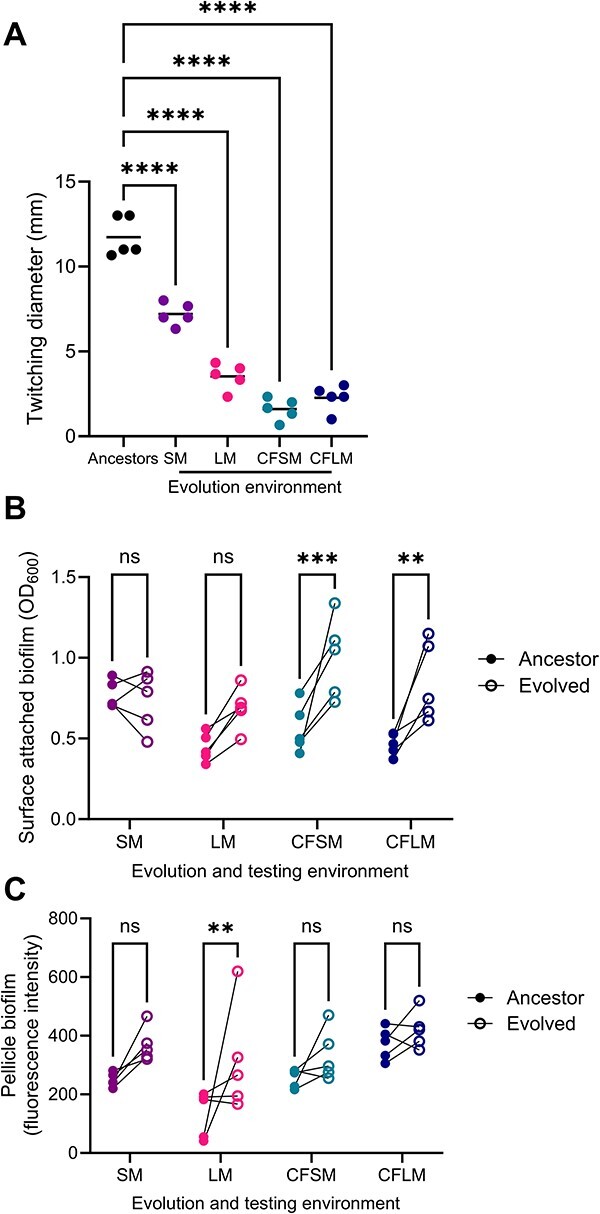
Loss of twitching motility and enhanced biofilm formation in airway-adapted PAO1; (A) endpoint (passage 20) populations from each of the experimentally evolved populations were stab inoculated onto LB agar, and after overnight growth at 37°C, crystal violet staining was used to visualize and quantify the diameter of bacterial growth; each data point represents an individual population and is the mean of three independent experiments; statistical analysis is by one-way ANOVA with Dunnett’s multiple comparison test, with the ancestor PAO1 set as the comparator group; ^*^^*^^*^^*^ = *P* < .0001; (B) surface attached and (C) pellicle biofilm formation by endpoint populations and the individual PAO1 colonies from which they were evolved; biofilm mass was determined by (B) crystal violet staining or (C) resazurin-determined quantification of metabolic activity from 72-h cultures; each population was tested in the media within which it had been evolved; ancestors were tested separately in each media, and lines link each evolved population to the ancestor from which it was derived; *P*-values were determined by two-way ANOVA with Sidak’s multiple comparison test, comparing evolved populations to ancestors within each test media; ^*^^*^ = *P* < .01, ^*^^*^^*^ = *P* < .001.

A striking feature of the variants identified by population sequencing analysis of airway-adapted PAO1 was the prevalence of mutations in genes encoding type IV pili (T4P) components (Table 1). These included genes involved in pilus assembly (*pilB, pilN*), retraction (*pilT*) and secretin channel formation (*pilQ)*, as well as the minor pilin *pilE*. However, few of the identified mutations were fixed in individual populations and the changes included non-sense mutations, non-synonymous single nucleotide polymorphisms (SNPs), deletions, and changes in intergenic regions. To determine the collective impact of these changes, we quantified twitching motility—a T4P-dependent phenotype—in airway-adapted populations (Fig. 3A). Significant twitching impairment was apparent across all populations but was most pronounced in those evolved in the CF-like environments (one-way ANOVA, *P* < .0001, *F* = 132.3, DF = 4, 20).

**Table 1:** Variants in type IV pili and cyclic-di-GMP regulation genes in PAO1 evolved under airway-mimicking conditions; variants were identified with Breseq; locus tags are from PAO1 (GCF_000006765.1), and position is relative to the origin of replication.

**Gene**	**Locus**	**Function**	**Mutation**	**Mutation Class**	**Position**	**Population**	**Frequency (%)**
*siaA*	PA0172	biofilm regulation protein phosphatase	G → A	Intergenic	196 835	**CFLM 3**	78
			G → A	Intergenic	196 837	**CFLM 4**	44
*bifA*	PA4367	cyclic di-GMP phosphodiesterase	∆4 bp	Deletion	4 894 798	**SM 2**	5
			Q306^*^	Nonsense SNP	4 895 374	**SM 2**	46
			∆33 bp	Deletion	4 895 962	**SM 3**	92
			W462^*^	Nonsense SNP	4 895 843	**SM 5**	14
			Q463^*^	Nonsense SNP	4 895 845	**LM 1**	69
			∆4 bp	Deletion	4 894 845	**LM 2**	90
			E551K	Non-syn SNP	4 896 109	**LM 4**	59
			Q226^*^	Nonsense SNP	4 895 254	**LM 5**	32
			Q302^*^	Nonsense SNP	4 895 362	**LM 5**	100
*dipA*	PA5017	cyclic di-GMP phosphodiesterase	I703T	Non-syn SNP	5 643 117	**CFSM 1**	48
			E310^*^	Nonsense SNP	5 641 937	**CFSM 2**	19
			∆6 bp	Deletion	5 642 857	**CFSM 3**	67
			∆6 bp	Deletion	5 643 154	**CFSM 5**	61
			Q413^*^	Nonsense SNP	5 642 246	**CFLM 1**	10
			∆6 bp	Deletion	5 642 857	**CFLM 1**	14
			∆6 bp	Deletion	5 642 857	**CFLM 4**	12
			∆18 bp	Deletion	5 642 807	**CFLM 5**	17
*pilB*	PA4526	type IV fimbrial biogenesis protein	R398H	Non-syn SNP	5 070 955	**LM 1**	39
			Q552^*^	Nonsense SNP	5 071 416	**LM 1**	17
			E476^*^	Nonsense SNP	5 071 188	**CFSM 4**	11
			D388A	Non-syn SNP	5 070 925	**CFLM 3**	73
*pilE*	PA4556	minor type IV pilin protein	T117P	Non-syn SNP	5 104 862	**LM 1**	31
*pilN*	PA5043	type IV fimbrial biogenesis protein	∆13 bp	Deletion	5 679 577	**CFLM 1**	81
			∆13 bp	Deletion	5 679 577	**CFLM 3**	21
			∆13 bp	Deletion	5 679 577	**CFLM 5**	13
*pilQ*	PA5040	type IV fimbrial biogenenesis outer membrane protein	T605P	Non-syn SNP	5 676 046	**SM 2**	6
			∆18 bp	Deletion	5 676 088	**SM 2**	24
			T605P	Non-syn SNP	5 676 046	**SM 4**	100
			∆1 bp	Deletion	5 676 921	**CFSM 1**	9
			∆1 bp	Deletion	5 676 142	**CFSM 5**	100
			∆18 bp	Deletion	5 676 088	**CFLM 2**	100
			I452S	Non-syn SNP	5 676 504	**CFLM 4**	68
			∆1 bp	Deletion	5 676 142	**CFLM 5**	74
*pilT*	PA0395	twitching motility protein	∆12 bp	Deletion	437 139	**SM 1**	8
			∆15 bp	Deletion	437 539	**SM 2**	10
			H166N	Non-syn SNP	437 065	**SM 5**	45
			∆12 bp	Deletion	437 139	**LM 4**	46
			A288V	Non-syn SNP	437 432	**LM 4**	31
			L256H	Non-syn SNP	437 336	**CFSM 5**	13
			P197L	Non-syn SNP	437 159	**CFSM 3**	19

^*^indicates the introduction of a premature stop codon.

Non-syn, non-synonymous; bp, base pair.

SM and LM are sinus media and lung media, respectively. The CF prefix indicates supplementation of media with cystic-fibrosis media components. Frequency represents the prevalence of the identified gene variant in the total population.

Many airway-adapted populations acquired mutations in genes involved in cyclic-di-GMP signalling (Table 1). Deletions in the cyclic-di-GMP phosphodiesterase-encoding gene *dipA* were identified in two CFSM-adapted populations and one CFLM-adapted population, with two other CFSM-adapted populations carrying *dipA* SNPs (non-sense and non-synonymous) (Table 1). Deletions, non-sense mutations, and non-synonymous SNPs were also identified in a second phosphodiesterase gene, *bifA*, in three SM-adapted and four LM-adapted populations. Finally, two CFLM-adapted populations acquired SNPs in the −10 site of the promoter of *siaA*, which encodes a phosphatase that regulates cyclic-di-GMP signalling via phosphorylation of the SiaC component of the SiaC-SiaD diguanylate cyclase complex [24].

**Figure 4:**
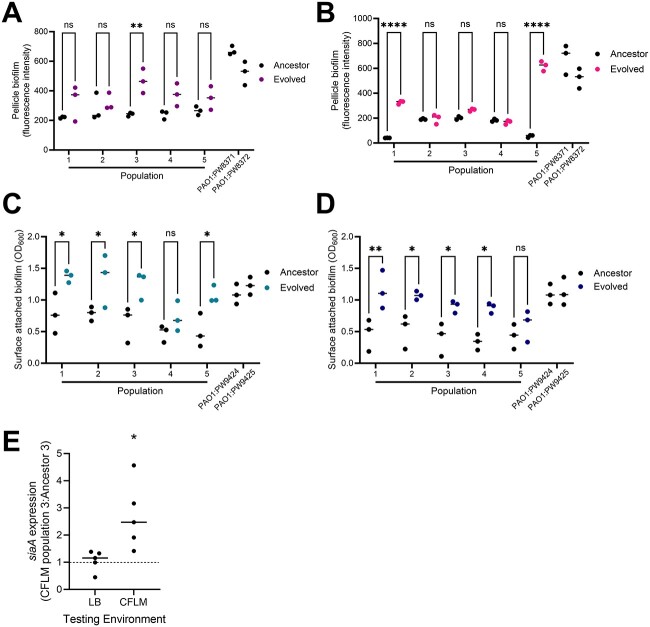
Association of mutations in genes involved in cyclic-di-GMP regulation with biofilm phenotypes; pellicle (A, B) and surface-attached (C, D) biofilm formation by individual populations evolved within and tested in (A) SM, (B) LM, (C) CFSM, and (D) CFLM; each population was compared to its respective ancestor in the same media; each data point represents the mean of one biological replicate. Four transposon mutants from the PAO1 transposon library were included in these assays: PAO1:PW8371, PAO1:PW8372 (transposon insertions in *bifA*), and PAO1:PW9424, PAO1:PW9425 (transposon insertion in *dipA*); *P*-values were determined by two-way ANOVA with Sidak’s multiple comparison test, comparing evolved populations to their respective ancestors; Ns = *P* > .05, ^*^ = *P* < .05, ^*^^*^ = *P* < .01, ^*^^*^^*^^*^ = *P* < .0001 for pairwise comparisons; (E) expression of *siaA* in CFLM-evolved population 3, relative to its PAO1 ancestor when grown in LB and CFLM; expression was determined by qRT-PCR and analyzed using the 2^-ΔΔCt^ method, with *rpoD* as the housekeeping gene; each data point is the mean of individual biological replicates. ^*^ = *P* < .05 in two-tailed *T*-test with Welch’s correction vs ancestor 3.

Cyclic-di-GMP regulates important cellular processes, including surface colonization and biofilm formation [25]. We quantified the ability of airway-adapted populations to form both surface attached biofilms (Fig. 3B) and free-floating aggregates (Fig. 3C). These assays were performed in the media within which each population had been evolved. Those passaged in CF-like media showed enhanced surface-attached biomass under those same conditions (Fig. 3B) (two-way ANOVA, population: *P* < .0001, *F* = 24.8, DF = 1, 32, environment: *P* = .0240, *F* = 3.6, DF = 3, 32), while increased aggregate formation was apparent only in populations evolved within non-CFLM (Fig. 3C) (two-way ANOVA, population: *P* = .0004, *F* = 15.3, DF = 1, 32, environment: *P* = .0011, *F* = 6.8, DF = 3, 32). At the level of the individually evolved populations, enhanced aggregate formation was identified in those carrying mutations in cyclic-di-GMP regulating genes. SM-evolved population 3 (33 base pair deletion in *bifA*, at 92% frequency) and LM-evolved populations 1 and 5 (non-sense *bifA* mutations at 69% and 100% frequency, respectively) showed enhanced pellicle biofilm formation, relative to the ancestor PAO1 colonies from which they were derived (Fig. 4A,B). PAO1 carrying a transposon insertion in *bifA* (PAO1:PW8371, PAO1:PW8372) showed a similar phenotype (Fig. 4A,B). LM-evolved population 2 (4 base pair deletion in *bifA*, at 90% frequency) was the only population harbouring a high frequency *bifA* mutation that showed no apparent change in free-floating biofilm formation.

CFSM-evolved populations 1, 2, 3, and 5, each of which carried mutations in the *dipA* phosphodiesterase, showed no evidence of increased propensity to form aggregates (Supplementary Fig. 4, see online supplementary material for a colour version of this figure) but displayed enhanced surface-attached biofilm formation, as did PAO1 with a transposon insertion in *dipA* (PAO1:PW9424, PAO1:PW9425) (Fig. 4C). CFSM population 1 harboured an SNP in *dipA*, at 48% frequency. CFSM population 2 had a *dipA* nonsense mutation at 19% frequency, and populations 3 and 5 each contained unique six base-pair deletions, present at 67% frequency. CFLM-evolved population 4 harboured a *dipA* six base-pair deletion and an SNP in the predicted −10 site of the *siaA* phosphatase promoter. The same *siaA* mutation was also observed in CFLM-evolved population 3. Both populations showed enhanced surface-attached biofilm formation, relative to their ancestors (Fig. 4D). To determine whether the promoter SNP might influence this phenotype, we quantified *siaA* expression in CFLM population 3. We observed environment-dependent increases in *siaA* expression in the evolved population, relative to the ancestor, with little difference in expression between the two populations when grown in standard laboratory media (two-tailed *t*-test with Welch’s correction vs ancestor, *P* = .7312, DF = 4) but significantly increased expression in the adapted PAO1 when grown in CFLM (two-tailed *t*-test with Welch’s correction vs ancestor, *P* = .0359, DF = 4) (Fig. 4E).

**Figure 5:**
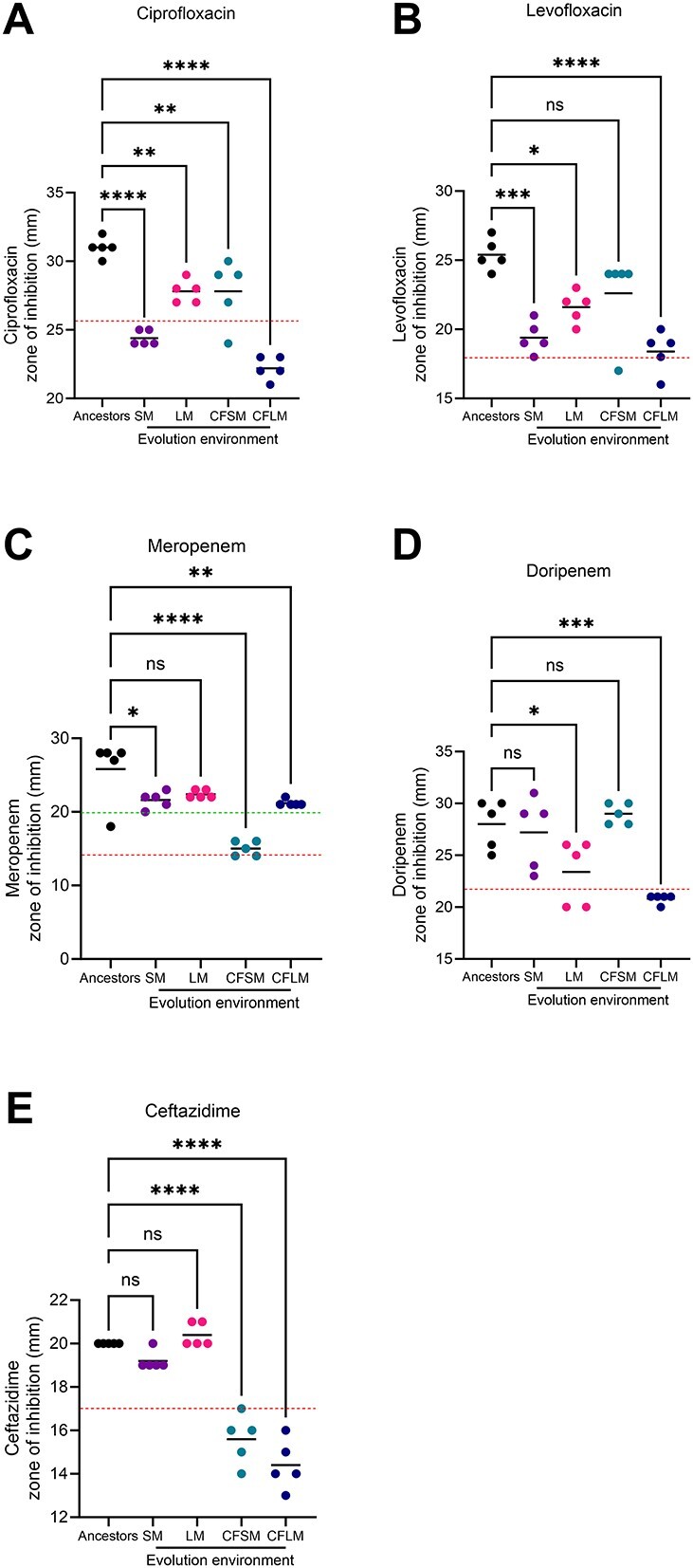
Increased antimicrobial resistance in airway-adapted PAO1; antimicrobial resistance against (A) ciprofloxacin, (B) levofloxacin, (C) meropenem, (D) doripenem, and (E) ceftazidime was determined by disc diffusion assay; each data point represents an independently evolved population (SM, LM, CFSM, CFLM) or the five ancestral PAO1 colonies from which they were derived (Ancestors); each data point is the mean of three independent biological experiments, and dashed lines represent clinical breakpoints for sensitivity (upper line in Meropenem plot) or resistance (all other dashed lines), as defined by EUCAST (v13.1); cut-offs for ciprofloxacin, levofloxacin, doripenem, and ceftazidime sensitivity are 50 mm; *P*-values were determined by one-way ANOVA with Dunnett’s multiple comparison test, with the ancestor set as the comparator column; Ns = *P* > .05, ^*^ = *P* < .05, ^*^^*^ = *P* < .01, ^*^^*^^*^ = *P* < .001, ^*^^*^^*^^*^ = *P* < .0001 for pairwise comparisons.

Although no antibiotics were added to cultures during the experimental evolution process, previous studies suggest that certain ecological contexts select for traits conferring antimicrobial resistance or susceptibility, independently of antimicrobial exposure [11, 26]. We quantified resistance to five antimicrobials in evolved populations, using disc diffusion assays (Fig. 5). We observed significant increases in resistance of the evolved populations to fluoroquinolones (Fig. 5A,B), carbapenems (Fig. 5C,D), and a cephalosporin (Fig. 5E) (one way ANOVA, DF = 4, 20 for all, ciprofloxacin *P* < .0001, *F* = 36.7, levofloxacin *P* < .0001, *F* = 11.9, meropenem *P* < .0001, *F* = 17.4, doripenem *P* = .0001, *F* = 10.4, and ceftazidime *P* < .0001, *F* = 60.3). In some cases, these changes resulted in populations crossing clinical breakpoints for resistance. The populations evolved under CF lung-like conditions displayed the highest level of resistance to 4 of the 5 agents tested.

## Discussion

*Pseudomonas aeruginosa* establishes chronic infections across a spectrum of respiratory disorders, including CF, NCFB, and COPD. Understanding of *P. aeruginosa* adaptation and evolution within the CF lung has progressed significantly [2], aided by a patient community that is familiar and comfortable with participation in research, but also by efforts from across the research community to develop laboratory models reflective of CF airway conditions. Sputum mimics, capturing the chemical and physical properties of CF sputum, have been extensively used in the study of CF infection [17, 27–29]. Comparable models for study of COPD and NCFB are scarce, and less is known about how adaptive evolutionary processes play out in non-CF airway environments. Broad clinical definitions of COPD and NCFB have made defining the associated respiratory environments challenging, although there has been notable recent progress [30–32].

To expand the pool of available sputum mimics, we included the representation of upper airway environments and non-CF respiratory conditions [17]. We used those media to compare *P. aeruginosa* adaptations across different airway contexts. Our findings highlight multiple evolutionary routes to the emergence of generalizable airway adaptations. Biofilm formation, loss of motility, wrinkly colony morphotypes, and increased AMR were features of PAO1 evolved within each of the four respiratory environments. The wrinkly colony phenotype is a common adaptation to respiratory niches, aiding in oxygen and nutrient transport across biofilm surfaces [33–35]. Its appearance under all four conditions reinforces the notion that both upper and lower respiratory tract select for biofilm modes of growth.

Although motility, AMR, and morphology phenotypes associated with *P. aeruginosa* adaptation to CF airways were observed in our study, we saw little evidence of the slow growth that has been described as a feature of CF isolates [36–38]. Longitudinal CF isolate sampling has demonstrated that there are multiple evolutionary routes to airway persistence, not all of which are associated with slow growth [39]. The short duration of the evolution study undertaken here might have been insufficient for growth-attenuated mutants to emerge. Alternatively, if altered growth phenotypes were environment-specific then they may have been missed, as growth rate determination was performed in LB. However, the slow growing phenotype of *P. aeruginosa* isolates from CF is observable in nutrient broth [37]. The minor growth phenotypes that were observed here, of increased productivity but decreased carrying capacity in SM-evolved populations and decreased generation time in LM-evolved populations, were not readily explainable by the presence of fixed mutations in those populations. Subpopulations carrying low-frequency mutations conferring improved substrate uptake and utilization or decreased production of metabolically costly resources might have outcompeted other clones within each population. Follow-up studies will be required to investigate this possibility.

The strength of selection differed between CF and non-CF niches. Populations evolved under CF sinus or CF lung-like conditions were the least motile, formed the most robust surface-attached biofilms, and showed the most consistent increases in resistance to multiple classes of antimicrobials. Emergence of these traits under CF conditions is in line with previous observations in sputum mimics and with clinical isolates [40–43]. Despite the more pronounced phenotypic changes under CF conditions, the populations evolved under non-CF airway conditions showed the greatest increases in environment-specific fitness during competition experiments. This might reflect a limit to the achievable fitness (that is, growth rate), under CF conditions, within the experimental timeframe. CF media contain high concentrations of host-derived antimicrobials and other stress factors that limit growth. Evolved populations from all four conditions showed evidence of increased colonization potential in a respiratory infection mouse model, albeit in a non-diseased airway context. Comparison of relative fitness of populations in a CF airway infection model could provide further insights into environment-specific adaptations.

Chemical second messengers play important roles in bacterial biological processes, including surface attachment and virulence [44]. Mutations in genes encoding products involved in second messenger signalling were frequently observed in this study. Type IV pili (T4P) mediate surface attachment and twitching motility and act as mechanochemical stimuli for cAMP production. *Pseudomonas aeruginosa* lacking the ability to retract T4P (*pilT* mutants) and those unable to form functional pili (those lacking the PilB ATPase) are attenuated in cAMP signalling and have a consequent defect in virulence traits, including quorum sensing (QS) and type II and III secretion that are regulated by the cAMP-responsive transcription factor Vfr [45]. We identified mutations in *pilB*, *pilE*, *pilN*, *pilQ*, and *pilT* and found that all four respiratory environments selected for loss of twitching motility. Non-motile phenotypes are frequently observed in isolates from chronic respiratory infection [41]. Although T4P are important for initial surface attachment, bacteria that are horizontally oriented across a surface are more likely to remain attached if they lack type T4P, a phenomenon ascribed to the propensity for pili to pull cells toward a vertical orientation, facilitating detachment [46]. Thus, mutants lacking T4P function tend to form biofilms that are patchy, due to impaired mobility, but also high-density, due to impaired detachment [47]. Consistent with this, experimentally evolved populations bearing *pil* mutations accumulated more biomass in surface attached biofilm assays.

The role of T4P mutations in adaptation to the airways is unclear. The association of particular T4P alleles with CF isolates suggests that pili might contribute to fitness in the airways [48], and it has been suggested that loss of minor pilins might be adaptive due to the downstream effects of alleviating feedback inhibition on FimS-AlgR and thereby reducing virulence factor expression [49]. However, T4P mutations are observed in experimental evolution studies conducted under a wide variety of environmental conditions [50–53], and so, their appearance in this study is not necessarily indicative of an airway-adapted phenotype.

T4P act as indirect regulators of cyclic-di-GMP signalling. Through stimulation of cAMP-dependent Vfr activation, T4P lower intracellular c-di-GMP via a mechanism that involves two c-di-GMP phosphodiesterases, DipA and BifA [54]. We identified multiple loss of function mutations in *dipA* and *bifA*, suggesting that the disruption of this negative feedback loop that might achieve decoupling of cAMP and c-di-GMP signalling.

When *P. aeruginosa* encounters a surface, intracellular levels of c-di-GMP rapidly accumulate and promote gene expression signatures associated with tissue adherence, biofilm formation, and virulence [55]. Once surface attachment is achieved, asymmetric cell division leads to daughter cells with opposing functionality, resulting from their differing c-di-GMP levels. The cell with high c-di-GMP is adherent, while the low c-di-GMP is flagellated and geared toward dispersal [56]. While this process is important in early colonization, c-di-GMP plays an equally critical role in established chronic infections, mediating surface exploration, the formation of microcolonies and eventually biofilms [55].

Regulation of c-di-GMP signalling involves the coordination of the activities of the diguanylate cyclases, which synthesize the second messenger, and phosphodiesterases that promote its degradation. Loss of BifA phosphodiesterase activity leads to a hyperbiofilm phenotype, due to reduced capacity of lower intracellular c-di-GMP [57]. We observed the loss of function mutations in *bifA* under non-CF conditions and mutations in another phosphodiesterase, *dipA*, in populations evolved in CF sinus or LM. The genetic switch controlling *P. aeruginosa* surface colonization is mediated by inhibition of BifA, triggered through HecE activity under conditions of nutrient limitation or elevated temperature [55]. Loss of BifA function in airway-adapted *P. aeruginosa* might fix cells into a regulatory mode geared toward surface-attachment and a sessile lifestyle. A high frequency of non-sense mutations in *bifA* has been reported in *P. aeruginosa* isolated from NCFB [1].

We identified SNPs proximal to the transcriptional start site of *siaA*, encoding a phosphatase that removes inhibitory phosphate groups from SiaC, promoting diguanylate cyclase c-di-GMP synthesis [24]. The SNPs changed the predicted *siaA* promoter −10 site from CACAAT to CA**T**AAT (CFLM population 3) or **T**ACAAT (CFLM population 4), in both cases bringing the sequence nearer to the sigma-70 promoter consensus −10 sequence (TATAAT). We observed increased expression of *siaA* in CFLM population 3, suggesting that the SNP might facilitate more efficient RNA polymerase holoenzyme binding. The upregulation was environment-specific, with a more pronounced increase under CF-like conditions than in standard bacterial growth media (LB). Follow-up studies will determine how environmental sensing might regulate expression at this locus. We also observed multiple low-frequency mutations in *wsp* genes under all airway conditions (*wspABCEF*) (Supplementary Table 1). These loci have been implicated in the regulation of biofilm formation and cyclic-di-GMP production [58], and mutations have been recorded in CF isolates [59].

Aside from the clear evidence of selection for mutations in second messenger signalling systems, we observed some low-frequency mutations in other loci that have been associated with *P. aeruginosa* adaptation to infection-relevant conditions in previous experimental evolution studies [52, 60]. Genes associated with iron uptake were frequently mutated, including pyoverdine (*pvd*) and pyochelin (*pch*) genes, and an iron transporter (*feoB*). These mutations arose under all experimental conditions and were generally found at low frequencies (Supplementary Table 1). Low-frequency mutations in *pvd* genes, perhaps indicative of social cheats, have been observed in host environments of low spatial structure, with higher frequencies of mutation at these loci only sustainable in the absence of host factors [52].

The emergence of hypermutator and mucoid phenotypes have been frequently associated with adaptation in *P. aeruginosa*, including in a CF airway context [59, 61]. We did not directly assess these phenotypes, but the associated mutations were rare. We identified a non-synonymous SNP in *mutS* in one CFLM-evolved lineage and a synonymous SNP in *mutL* in an SM-evolved lineage. Mismatch repair gene mutations in *mutS* and *mutL* are the most common causes of hypermutability in *P. aeruginosa* isolates [62]. Similarly, there was little evidence of selection for mucoidy in the airway-mimicking media, with only two low-frequency mutations identified in *mucB*, encoding a negative regulator of the AlgU alternative sigma factor that controls alginate biosynthesis [63]. Mutations in *mucB*, and more commonly *mucA*, that confer a mucoid phenotype are a feature of *P. aeruginosa* isolates from CF [59, 64, 65].

Mutations in QS loci are common amongst airway *P. aeruginosa* isolates [59, 66]. The success of clones carrying such mutations derives from social exploitation of QS signals and therefore requires their coexistence with QS wild-type lineages, limiting the frequency of QS mutants that can be supported in a population [67]. We observed no *lasR* mutations under any of the airway-mimicking conditions and found only a single mutation proximal to a QS locus, in an intergenic region between *rsaL* and *lasI*. This contrasts with the high frequency of QS mutations observed in other experimental evolution studies [52, 60, 68].

Increased resistance to multiple classes of antimicrobials was observed in populations evolved within airway-mimicking media, especially those evolved under CF-like conditions. No antibiotics were used during passage and so this resistance results from other environmental adaptations or else may be driven by host-derived antimicrobials included in all four respiratory media and that are at higher concentrations in CF media [17]. Antibiotic-independent drivers of resistance evolution are recognized as important contributors to the AMR crisis [26, 69–71]. The combination of the altered airway environment and intensive antimicrobial chemotherapy in people with CF may create the perfect storm for resistance emergence. Of note, c-di-GMP contributes to antimicrobial resistance through biofilm-dependent and biofilm-independent mechanisms [72]. Both overexpression of a diguanylate cyclase gene (*PA5487*) and lowering of c-di-GMP through *sagS* deletion confer antimicrobial susceptibility [73, 74]. In the case of Δ*sagS*, this phenotype is not explained by reduced biofilm formation alone [74]. The extent to which the hyperbiofilm phenotype contributes to the resistance observed here is unclear. Disc diffusion assays were performed according to EUCAST diagnostic methodology, over 18 h of culture. There is an opportunity for biofilm formation to contribute to resistance during this time, but the strongest biofilm formers were not necessarily the most resistant populations. We observed no fixed mutations in genes associated with antimicrobial resistance, but lower frequency mutations within or proximal to efflux pump genes or regulators, including *mexEF*, *mexT and mexXY*, may contribute to the observed phenotypes.

Collectively, these data are in line with clinical observations of similarities in adaptive phenotypes of *P. aeruginosa* isolated from CF and other respiratory origins, including NCFB. We demonstrate that loss of twitching, increased biofilm, and increased AMR are features of adaptation to both upper and lower airway environments, under CF and non-CF conditions. A recurring feature of the evolutionary routes to adaptation was mutations in genes regulating second messenger signalling. This included both those directly promoting c-di-GMP signalling (*bifA, dipA, siaA*) and those that do so through inhibition of cAMP signalling (*pil* genes). Such mutations likely represent adaptations to a sessile lifestyle that might promote chronicity of infection at the expense of pioneering or environmental dispersal phenotypes.

## Supplementary Material

Supp_Figs_Dilem_ExpEvo_v1_wrae065

Supp_Table_1_R1_wrae065

## Data Availability

DNA sequence data are available at NCBI Bioproject PRJNA1049764. The remaining datasets generated during and/or analyzed during the current study are available from the corresponding author on reasonable request.
