# Supplementary material for: Secondary messenger signalling influences Pseudomonas aeruginosa adaptation to sinus and lung environments
Source: ISME J. 2024 Apr 22;18(1):wrae065. doi: 10.1093/ismejo/wrae065 (PMC11102083; doi:10.1093/ismejo/wrae065)
Supplement: Supp_Figs_Dilem_ExpEvo_v1_wrae065 [file supp_figs_dilem_expevo_v1_wrae065.docx]

**Secondary messenger signalling influences *Pseudomonas aeruginosa* adaptation to sinus and lung environments**

**Supplementary Figures**

**
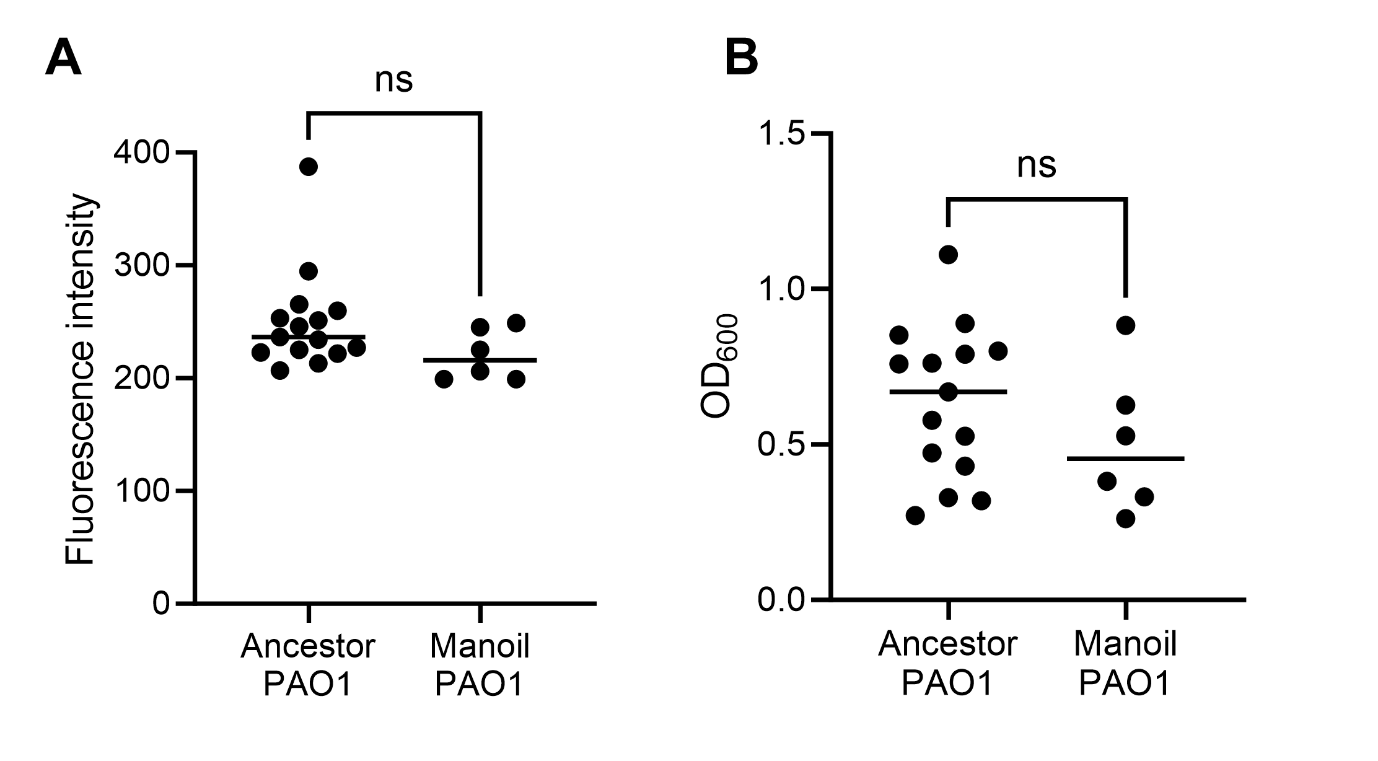
**

**Supplementary Figure 1. Biofilm formation by PAO1 isolates.** Formation of **(A)** pellicle and **(B)** surface-attached biofilms by the PAO1 isolate used for experimental evolution (ancestor) and the PAO1 with which the transposon library was generated (Manoil). Ns = not significant in Mann Whitney test.

**Supplementary Figure 2. Growth of airway-adapted lineages in LB.** Airway-adapted lineages and the ancestral PAO1 colonies from which they were evolved were profiled for growth in LB in 96-well plates. **(A)** lineages derived from PAO1 ancestor 1, **(B)** lineages derived from PAO1 ancestor 2, **(C)** lineages derived from PAO1 ancestor 3, **(D)** lineages derived from PAO1 ancestor 4, **(E)** lineages derived from PAO1 ancestor 5.


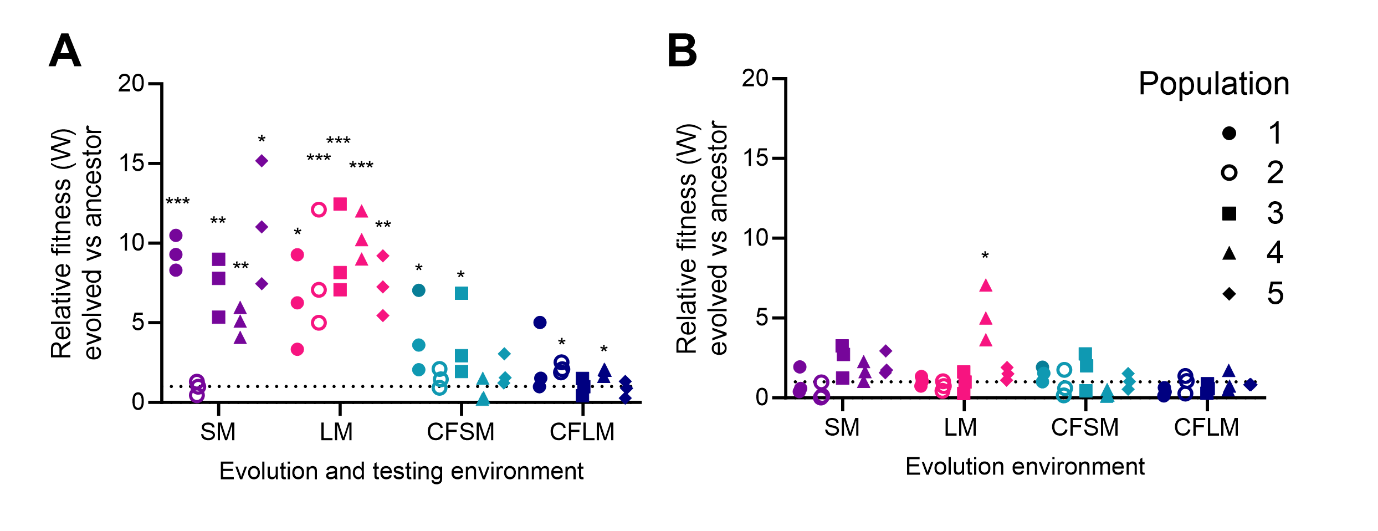


**Supplementary Figure 3. Environment-specific fitness in evolved populations.** Relative fitness of passage 20 populations vs PAO1 tagged with a gentamicin resistance cassette, determined by a 24 h competition assay using **(A)** the media in which each set of populations had been evolved, or **(B)** LB. For each environmental condition, the five separately evolved populations were competed independently against gentamicin-resistant PAO1. Relative fitness calculations were performed by calculating the Malthusian parameter (growth rate; m) for each competitor as ln(final density/starting density) and by taking the ratio between PAO1 and evolved populations (m_PAO1/m_population) to get a fitness coefficient (W). W>1 (above dashed line) represents enhanced fitness relative to the ancestor, under the conditions tested. Calculations were adjusted to account for the fitness disparity between PAO1 and gentamicin-resistant PAO1. Data are from a single experiment with three technical replicates per population. *’s represent significance vs W=1. * = p<0.05, ** = p<0.01, *** = p<0.001.

**Supplementary Figure 4. Pellicle biofilm formation in airway-adapted lineages.** Resazurin-determined quantification of metabolic activity from 72 hour cultures of **(A)** CFSM-evolved and **(B)** CFLM-evolved lineages, relative to the ancestor PAO1 from which they were derived. Each lineage was tested in the media within which it had been evolved. Each data point represents the mean of one biological replicate. P values were determined by two-way ANOVA with Sidak’s multiple comparison test, comparing evolved populations to their respective ancestors. Ns = p>0.05, ** = p<0.01.
